# Comparative Patterns of Cuticular Hydrocarbon Diversity in Termites Across Castes and Nesting Life Types

**DOI:** 10.1002/ece3.74070

**Published:** 2026-08-09

**Authors:** V. Palma‐Onetto, J. Cabrera‐Riquelme, S. Ahmad

**Affiliations:** ^1^ Departamento de Química y Medio Ambiente Universidad Técnica Federico Santa María Valparaíso Chile; ^2^ Termitolab ‐ Termite Research Lab Universidade Federal do ABC São Bernardo do Campo Brazil; ^3^ Guangdong Key Laboratory of Animal Conservation and Resource Utilization Institute of Zoology, Guangdong Academy of Science Guangzhou China

**Keywords:** CHC, chemical ecology, cuticular hydrocarbons, isoptera, termites

## Abstract

Cuticular hydrocarbons (CHCs) are central to insect waterproofing and chemical communication, yet their diversity and comparative distribution in termites have not been synthesized comprehensively. Here, we present a systematic review of termite CHCs with emphasis on worker profiles, caste‐level comparisons, and descriptive ecological interpretation. The qualitative synthesis included 28 studies, whereas the filtered statistical dataset comprised 37 worker profiles representing 37 species. Across the reviewed literature, termite cuticles contained a recurrent but variable combination of n‐alkanes, n‐alkenes, alkadienes, alkatrienes, mono‐, di‐, and trimethylalkanes. Species‐level mapping showed that saturated and methyl‐branched hydrocarbons were broadly distributed across the sampled taxa, whereas unsaturated compounds were more unevenly represented across the dataset. Genus‐level synthesis showed that methyl‐alkane‐dominated, olefin‐dominated, and mixed worker profiles occur in different higher taxonomic groups within the sampled dataset. Quantitative comparison of selected worker profiles highlighted broad chemistry in *Mastotermes darwiniensis*, strong methyl‐branched dominance in *Coptotermes formosanus* , dimethyl‐rich profiles in *Nasutitermes corniger* and *N. ephratae* , and marked alkatriene dominance in *Parvitermes wolcotti*. Caste‐resolved studies showed that workers, soldiers, nymphs, and reproductives usually share the same broad CHC classes, with caste differentiation arising mainly through quantitative shifts, although reproductives in some taxa also express caste‐associated compounds. Exploratory ecological analyses identified associations between nesting life type and worker CHC composition: drywood/enclosed wood nesters showed fewer dimethylalkanes and more frequent alkatrienes than other life types; but these patterns could not be separated from broad family‐level phylogenetic structure. Overall, the available evidence suggests that termite CHC diversity reflects variation in the relative representation of a recurring chemical toolkit, with exploratory associations between worker CHC composition and nesting life type. Because the current dataset is limited and phylogenetic signal was not formally tested, these interpretations should be considered exploratory.

## Graphical abstract

Cuticular hydrocarbon (CHC) profiles in termites show strong ecological structuring across life type, climate, and caste, based on a comparative analysis of published datasets. Despite this ecological signal, phylogenetic patterns are weak, indicating high evolutionary lability relative to other social insects. These results suggest that termite CHC evolution is shaped more by ecological pressures than by phylogenetic constraint, with important implications for chemical communication and adaptation.

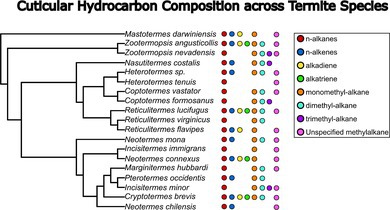

## 1 Introduction

Cuticular hydrocarbons (CHCs) are major components of the insect cuticle and represent one of the most multifunctional chemical traits in insects. Present across insect groups, they contribute to cuticular waterproofing and the reduction of transcuticular water loss (Gibbs and Pomonis 1995; Howard and Blomquist 2005; Menzel et al. 2019), can participate in pathogen defense (Howard and Blomquist 2005; Blomquist and Bagnères 2010), and mediate diverse recognition and communication processes, including species discrimination, mate choice, sexual selection, reproductive signaling, and social recognition (Johansson and Jones 2007; Blomquist and Bagnères 2010; Steiger and Stökl 2014; Blomquist and Ginzel 2021). For example, in non‐eusocial insects such as crickets and bush crickets, CHC profiles are associated with sexual selection, mate attraction, and species‐, sex‐, and mating‐status‐related variation (Thomas and Simmons 2009, 2010; Steiger et al. 2013; Sevgili et al. 2025). Nevertheless, CHCs have been studied especially intensively in eusocial insects, where they are central to nestmate recognition, caste differentiation, reproductive signaling, and colony‐level organization (Blomquist and Bagnères 2010; Menzel et al. 2019; Blomquist and Ginzel 2021).

In termites, CHC studies have historically accumulated through case‐specific investigations focused on taxonomy (Haverty et al. 1988, 1997, 2000; Haverty and Nelson 2005; Kaib et al. 2002), colony discrimination (Howard et al. 1978, 1982; Haverty et al. 1997), caste recognition (Howard et al. 1982; Sevala et al. 2000; Gordon et al. 2020), and fertility or reproductive signaling (Liebig et al. 2009; Weil et al. 2009; Funaro et al. 2018; Mitaka and Fujita 2021). These studies have revealed striking chemical diversity: some taxa are dominated by methyl‐branched hydrocarbons, others by unsaturated compounds, and still others by more compositionally balanced mixtures (Howard et al. 1978, 1982; Haverty et al. 1996, 1997, 2000; Haverty and Nelson 2005; Sevala et al. 2000; Aguilera‐Olivares et al. 2015). However, termite CHCs have not been synthesized within a broad comparative framework that connects this diversity to the wider insect chemical ecology literature while also evaluating whether termite hydrocarbon composition varies with taxonomic position, caste biology, and ecological context.

That question has become especially timely after the comparative Blattodea study of Golian et al. (2024), which showed that chemical divergence does not track molecular phylogeny or social complexity in a simple way. Their findings are particularly relevant to termites because the basalmost extant termite, *Mastotermes darwiniensis*, exhibits a broad and chemically mixed profile rather than an obviously primitive or simplified one, challenging any linear view of termite chemical evolution.

A second unresolved issue concerns caste differentiation. Several termite studies show that workers, soldiers, nymphs, and reproductives often share the same broad hydrocarbon repertoire, while differing in the relative abundance of particular compounds (Howard et al. 1982; Sevala et al. 2000). Other studies reveal reproductively enriched profiles or fertility‐linked compounds superimposed on an otherwise shared colony background (Weil et al. 2009; Liebig et al. 2009; Funaro et al. 2018; Mitaka and Fujita 2021). Together, these observations suggest that caste‐level chemical divergence may often be quantitative rather than qualitatively independent.

A third issue, and one that has been underappreciated in termite CHC discussions, is the potential role of ecology. Experimental work in *Cryptotermes brevis* showed that temperature and relative humidity can modify cuticular hydrocarbon composition (Woodrow et al. 2000). In other insects, climatic acclimation and habitat conditions are also known to influence the relative abundance of saturated, unsaturated, and branched hydrocarbons (Gibbs and Pomonis 1995; Menzel et al. 2017, 2019). This raises the possibility that termite CHCs may be shaped not only by ancestry but also by nesting life type, microclimatic buffering, and degree of exposure to external conditions.

Here, we present a systematic review of termite cuticular hydrocarbons with four objectives: (i) to compile the major hydrocarbon classes reported across termites and arrange them according to a published termite phylogenetic framework for descriptive visualization; (ii) to identify dominant worker CHC regimes at the genus level; (iii) to compare caste‐level patterns across the literature; and (iv) to quantify worker CHC composition in a subset of representative taxa and explore, in a hypothesis‐generating framework, whether life type, feeding group, and broad climate categories show descriptive associations with worker CHC profiles.

## 2 Materials and Methods

### 2.1 Systematic Review Design

This study was conducted as a systematic review of termite cuticular hydrocarbons following the general logic of PRISMA 2020. The review focused on primary studies reporting original data on cuticular or epicuticular hydrocarbons in termites, with emphasis on worker profiles and caste‐resolved comparisons. Study selection is summarized in Figure S1.

### 2.2 Literature Search Strategy

The literature search used combinations of general and taxon‐specific terms in English, including “termite cuticular hydrocarbons,” “termite epicuticular hydrocarbons,” “termite CHC,” “termite hydrocarbon profile,” “worker hydrocarbons termites,” “caste hydrocarbons termites,” and genus‐specific searches such as “*Reticulitermes* hydrocarbons,” “*Coptotermes* hydrocarbons,” “*Nasutitermes* hydrocarbons,” “*Zootermopsis* hydrocarbons,” and “*Mastotermes* hydrocarbons.” Searches were complemented by backward and forward citation tracking and by screening the project database.

In addition to studies explicitly focused on cuticular hydrocarbons, we also screened articles centered on termite gland chemistry when their experimental design included a control or comparison extract that allowed the cuticular hydrocarbon profile to be identified. Such studies were retained only when cuticular compounds could be distinguished clearly from glandular or other non‐cuticular fractions and extracted in a form comparable to the rest of the dataset.

### 2.3 Inclusion Criteria and Data Extraction

Studies were included when they reported original data on termite cuticular or epicuticular hydrocarbons, identified at least one hydrocarbon class from the cuticle, and provided species‐ or genus‐level taxonomic information. We extracted family, genus, species, caste, locality, climate, feeding group, life type, hydrocarbon classes, and relative abundance data when available. Worker profiles were prioritized for interspecific comparison. In taxa without a true worker caste, pseudergates or worker‐like immatures were treated as worker‐equivalent only when the original study used them as the main cuticular reference.

Compounds were standardized into the following classes: n‐alkanes, n‐alkenes, alkadienes, alkatrienes, monomethylalkanes, dimethylalkanes, trimethylalkanes, and unresolved branched hydrocarbons. The unresolved category was used only when the original study reported branched hydrocarbons without resolving them into subclasses.

### 2.4 Qualitative and Quantitative Datasets

Two datasets were built. The first was a qualitative matrix recording the presence or absence of major CHC classes across taxa. This matrix was used for the species‐level phylogenetic summary. Presence‐absence coding was used only for broad descriptive summaries of CHC class occurrence. Because this approach gives equal weight to trace and dominant compounds, binary data were not used to infer quantitative investment in particular hydrocarbon classes. Interpretations involving dominance, proportional shifts, or class‐level reweighting were based only on the worker quantitative matrix when relative abundance data were available. The second was a worker‐based quantitative matrix including taxa for which relative abundance by class could be extracted or calculated. This matrix was used for the genus‐level dominance summary and the comparison of selected worker profiles. The complete species‐by‐study matrix used to build the synthesis is provided in Table S1.

### 2.5 Ecological Variables

Taxa were assigned to four nesting categories: Drywood/enclosed wood nesters, Dampwood/wood‐dwelling, Subterranean/foraging, and Mound or external nest‐associated foragers. Feeding group was recorded as reported in the literature. Climate assignments were based on collection localities reported in the original publications and were subsequently grouped into broad climatic categories. These categories are unlikely to capture the actual microclimatic conditions experienced by termite colonies, particularly given the buffering effects of nest architecture and wood substrates.

### 2.6 Statistical Analyses

For statistical analyses, we retained only species for which worker CHC profiles were sufficiently resolved and directly comparable across studies. We excluded Batista‐Pereira et al. (2004) because the paper emphasized dominance patterns rather than a fully resolved class‐level worker profile, Mitaka and Fujita (2021) because it focused on caste‐associated compounds and did not provide a worker CHC profile comparable to the main matrix, and Nelson et al. (2008) because those studies were organized around chemical phenotypes and their correspondence with soldier defense secretions, making them valuable for qualitative interpretation but less directly comparable for species‐level ecological statistics.

The worker matrix was collapsed to one row per species, with class presence coded as present if reported in any qualifying worker study for that species. Fisher's exact tests were used for binary contrasts, chi‐square tests for multi‐category life‐type comparisons, and a Kruskal–Wallis test for class richness among life types.

As a descriptive sensitivity check for taxonomic imbalance, we repeated the main drywood versus non‐drywood Fisher's exact tests after excluding each family in turn. This procedure was not intended as a formal phylogenetic correction, but as a diagnostic assessment of whether the observed ecological contrasts were driven by the inclusion of particular termite families. We also tabulated life type by family to evaluate whether the ecological contrasts were confounded with broad phylogenetic structure. Because drywood/enclosed wood nesters were represented exclusively by Kalotermitidae in the filtered dataset, family‐adjusted models were not estimable.

The ecological analyses were intended as exploratory comparisons rather than formal tests of adaptive evolution. Because the dataset was assembled from heterogeneous literature sources and phylogenetic relationships were not incorporated into the statistical framework, species were treated as independent observational units for exploratory statistical analyses. Consequently, the results should be interpreted cautiously and primarily as hypothesis‐generating patterns.

## 3 Results

### 3.1 Study Inclusion and Scope of the Dataset

Qualitative synthesis comprised 28 studies that contributed usable information to this review. These studies documented cuticular hydrocarbons across a broad taxonomic range of termites and, in several cases, across multiple castes. Only a subset of papers provided class‐resolved quantitative data suitable for direct comparison, but the broader literature consistently reported the occurrence of major hydrocarbon classes at the species level. The complete coded dataset is provided in the Supporting Information.

Across the reviewed studies, termite cuticles contained a recurrent but highly variable combination of n‐alkanes, n‐alkenes, alkadienes, alkatrienes, monomethylalkanes, dimethylalkanes, and trimethylalkanes. However, both the presence and relative importance of these classes varied markedly among taxa, and the level of chemical resolution differed substantially among studies.

### 3.2 Distribution of Major CHC Classes Across Sampled Taxa Arranged Phylogenetically

**FIGURE 1:**
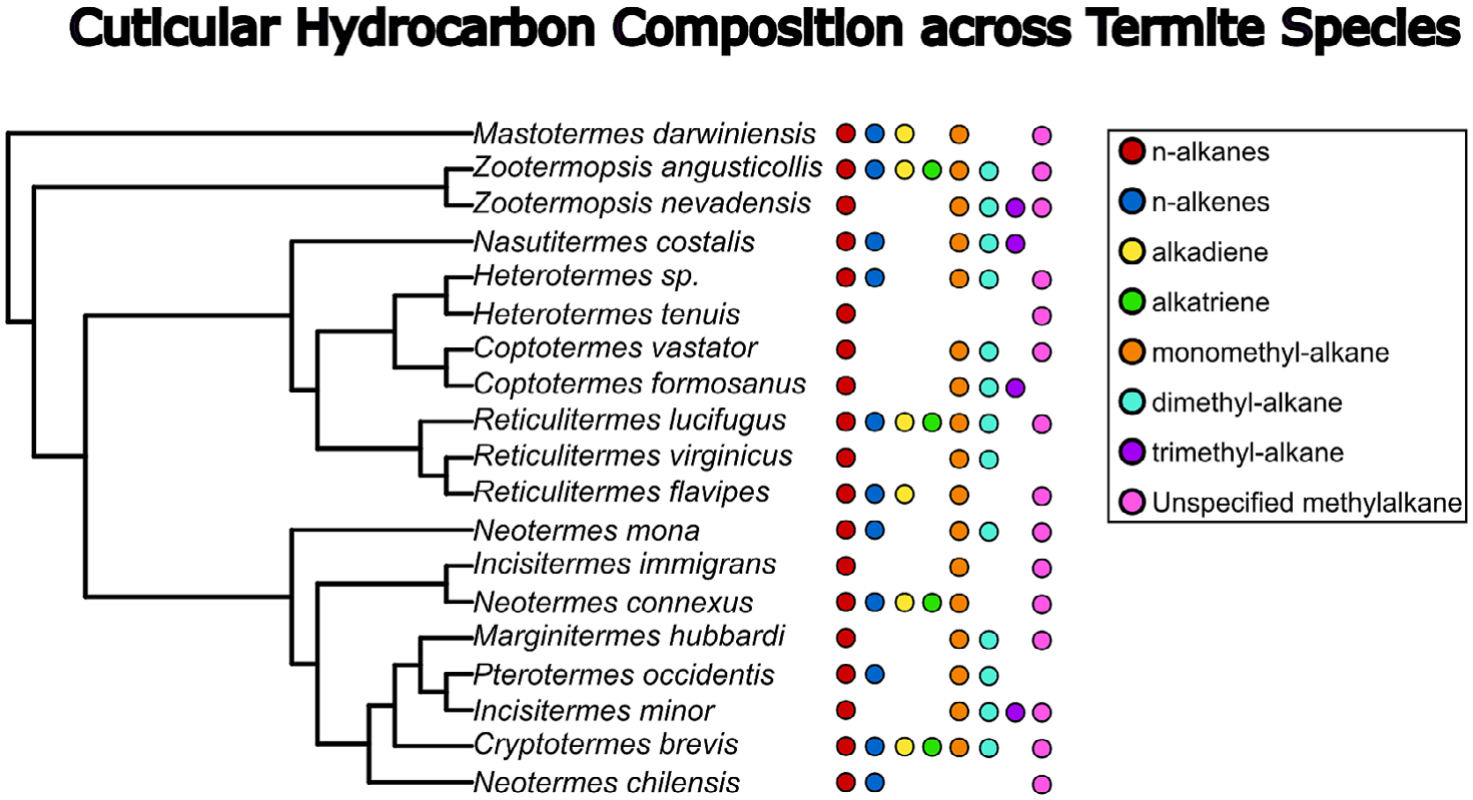
Species‐level arrangement of termites showing the presence or absence of major worker CHC classes. Taxa were arranged according to the termite phylogenetic framework of Buček et al. (2019). Symbols indicate the occurrence of n‐alkanes, n‐alkenes, alkadienes, alkatrienes, monomethylalkanes, dimethylalkanes, trimethylalkanes, and unresolved branched hydrocarbons. Worker profiles were used whenever available; worker‐equivalent immature stages were used only in taxa lacking a true worker caste.

The species‐level matrix showed that n‐alkanes and methyl‐branched hydrocarbons were broadly distributed among sampled termite taxa, whereas unsaturated compounds were more unevenly represented and occurred in different class combinations across the dataset (Figure 1). Some taxa contained only trace or low levels of unsaturated compounds, while others showed substantial contributions of alkenes, dienes, or trienes. Likewise, dimethyl‐ and trimethylalkanes varied strongly across the dataset and were not restricted to a single major termite clade.

At the basal end of the phylogeny, *Mastotermes darwiniensis* displayed one of the broadest worker profiles, including substantial fractions of saturated, unsaturated, and methyl‐branched hydrocarbons. Within Archotermopsidae, *Zootermopsis nevadensis* and *Z. angusticollis* were characterized mainly by n‐alkanes and branched hydrocarbons, although low or moderate unsaturated fractions were also reported depending on the species and study. In Kalotermitidae, chemical diversity was especially pronounced. *Neotermes connexus* combined n‐alkanes, monoenes, dienes, trienes, and monomethylalkanes in a broad worker profile, whereas *Neotermes mona* was more strongly biased toward methyl‐branched compounds. *Pterotermes occidentis* was dominated by n‐alkanes and monomethylalkanes, while *Cryptotermes brevis* , *C. cynocephalus* , *Incisitermes immigrans* , and *I. minor* retained substantial unsaturated fractions, including alkadienes and alkatrienes. By contrast, *Neotermes chilensis* and *Procryptotermes corniceps* were more strongly enriched in saturated and unresolved branched compounds.

Within Rhinotermitidae, *Coptotermes formosanus* , *C. vastator*, and *C. gestroi* were consistently dominated by methyl‐branched hydrocarbons and showed little or no contribution from unsaturated classes. *Heterotermes* was chemically more heterogeneous. The Australian species treated by Watson et al. (1989) showed profiles containing different combinations of alkenes, alkadienes, alkatrienes, and methyl‐branched hydrocarbons. In *Reticulitermes*, cuticular chemistry was consistently more complex. This was true not only for *R. flavipes* , *R. virginicus* , and *R. speratus*, but also for western European and Mediterranean taxa such as *R. santonensis* and *R. lucifugus*, in which n‐alkanes, monoenes, methyl‐branched compounds, and, in some species, dienes and trienes were all recorded.

Within Termitidae, strong variation in worker CHC composition was also evident. *Nasutitermes corniger* and *N. ephratae* were dominated by dimethylalkanes, whereas *Parvitermes wolcotti* showed one of the most extreme unsaturated profiles in the dataset, dominated by alkatrienes. *Nasutitermes acajutlae* retained a broad hydrocarbon repertoire but remained strongly biased toward unsaturated compounds.

### 3.3 Genus‐Level Patterns of Dominant Worker Chemistry

**FIGURE 2:**
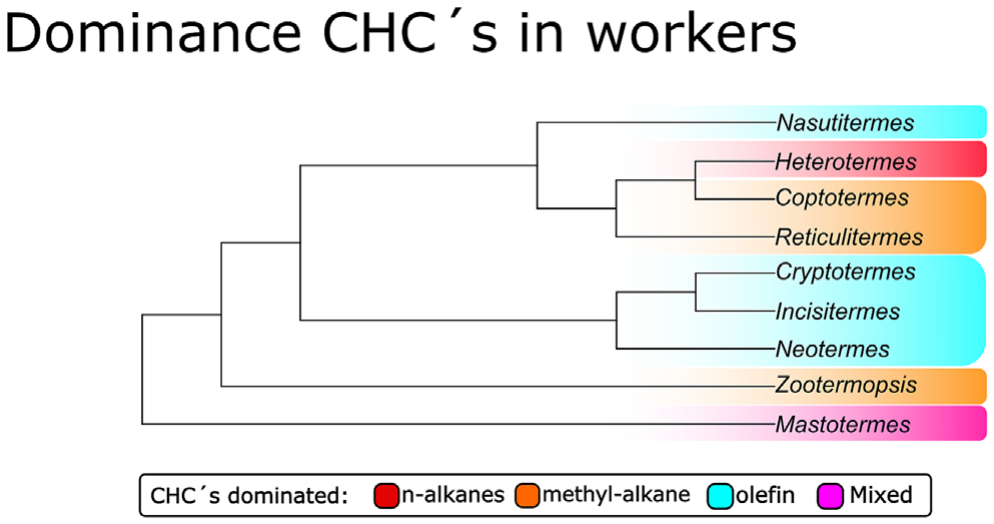
Genus‐level summary of dominant worker CHC chemistry across termites. Genera were classified as methyl‐alkane dominated, olefin dominated, or mixed according to the dominant worker compound class reported in the literature.

When worker profiles were summarized at the genus level according to their dominant hydrocarbon class, clear differences were observed among sampled genera (Figure 2). *Mastotermes* was best characterized as mixed, as its worker profile contained substantial fractions of saturated, unsaturated, and methyl‐branched hydrocarbons without a single overwhelmingly dominant class. *Zootermopsis* was predominantly methyl‐alkane dominated, reflecting the strong contribution of mono‐ and dimethylalkanes relative to unsaturated compounds. Within Kalotermitidae, dominant chemistry varied among genera: *Neotermes* and *Pterotermes* showed profiles ranging from mixed to olefin‐biased, whereas *Cryptotermes* and *Incisitermes* were more consistently associated with unsaturated‐rich worker blends. In Rhinotermitidae, *Coptotermes*, *Heterotermes*, and *Reticulitermes* were broadly consistent with a methyl‐alkane‐dominated condition, although the degree of unsaturated contribution differed among genera. Within Termitidae, marked contrasts were also evident, with *Nasutitermes* characterized mainly by methyl‐branched dominance, particularly dimethylalkanes, whereas *Parvitermes* was distinctly olefin dominated.

### 3.4 Relative Abundance Patterns in Selected Worker Profiles

**FIGURE 3:**
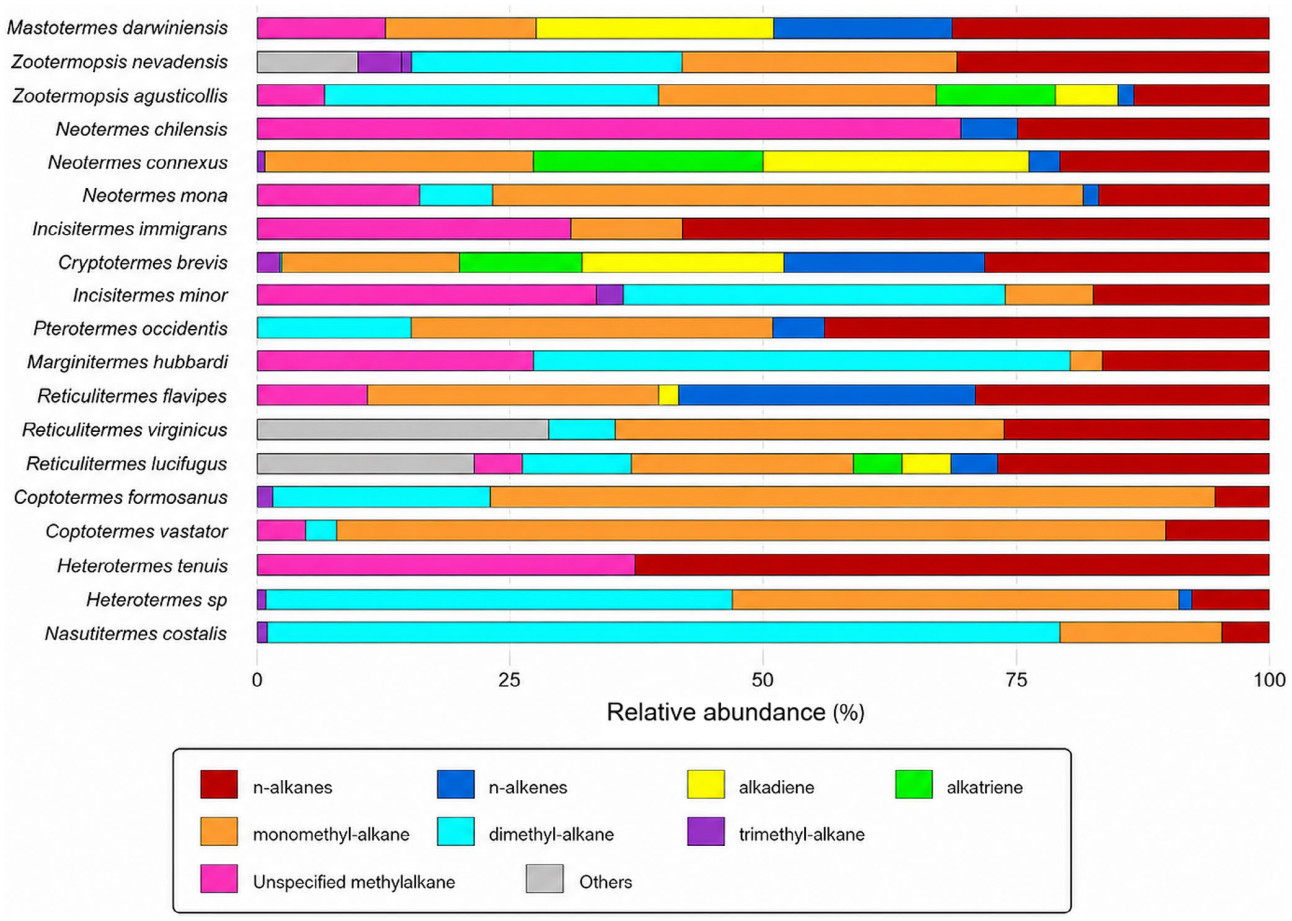
Relative abundance of major CHC classes in selected termite workers. Profiles are shown for available termite species. Bars represent relative proportions of n‐alkanes, n‐alkenes, alkadienes, alkatrienes, monomethylalkanes, dimethylalkanes, trimethylalkanes, and others.

The quantitative comparison of selected worker profiles highlighted strong interspecific variation in class‐level investment (Figure 3). *Mastotermes darwiniensis* displayed one of the most compositionally balanced profiles, with substantial fractions of n‐alkanes, n‐alkenes, alkadienes, and monomethylalkanes. *Zootermopsis nevadensis* was dominated by n‐alkanes, monomethylalkanes, and dimethylalkanes, with only low evidence of olefinic compounds in the full compound list. Within Kalotermitidae, *Neotermes connexus* combined n‐alkanes, monoenes, dienes, trienes, and monomethylalkanes in a broad profile, whereas *Neotermes mona* was shifted toward monomethylalkane dominance. *Pterotermes occidentis* combined high proportions of n‐alkanes and monomethylalkanes with a comparatively minor unsaturated fraction.

Within Rhinotermitidae, *Coptotermes formosanus* showed one of the most polarized worker profiles, dominated almost entirely by monomethylalkanes and lacking a meaningful unsaturated component. *Heterotermes* sp. was also dominated by methyl‐branched hydrocarbons, especially mono‐ and dimethylalkanes. *Reticulitermes flavipes* retained a more compositionally mixed worker profile in which n‐alkanes, n‐alkenes, and monomethylalkanes each contributed substantial fractions. Within Termitidae, *Nasutitermes corniger* and *N. ephratae* were strongly dominated by dimethylalkanes, whereas *Parvitermes wolcotti* differed sharply in being overwhelmingly dominated by alkatrienes.

### 3.5 Caste‐Level Comparisons Across the Reviewed Literature

The caste‐resolved literature showed that chemical differences among castes were common, but in most cases these differences reflected changes in relative abundance rather than complete replacement of the major CHC classes (Table 1). In *Reticulitermes virginicus* , workers, soldiers, nymphs, and reproductives shared the same main classes, including n‐alkanes, methylalkanes, dimethylalkanes, alkenes, and dienes, while caste differences were expressed primarily through proportional shifts (Howard et al. 1982). A similar pattern was reported for *Zootermopsis nevadensis* , in which castes shared the same general hydrocarbon repertoire, although the abundance of major compounds varied among workers and reproductive forms (Sevala et al. 2000). Comparable caste‐associated quantitative differences were also reported in *Coptotermes formosanus* and *C. gestroi* .

**TABLE 1:** Qualitative caste‐level CHC patterns reported in termites.

Taxon	Shared major CHC classes across castes	Quantitative differences among castes	Reproductive‐specific compounds or profiles reported	References
*Reticulitermes virginicus*	X	X		Howard et al. (1982)
*Zootermopsis nevadensis*	X	X	X	Sevala et al. (2000); Liebig et al. (2009)
*Nasutitermes acajutlae*	X	X		Haverty et al. (1996)
*Reticulitermes flavipes*	X	X	X	Funaro et al. (2018)
*Cryptotermes secundus*	X	X	X	Weil et al. (2009)
*Coptotermes formosanus*	X	X		Haverty et al. (1996)
*Coptotermes gestroi*	X	X		Gordon et al. (2020)
*Reticulitermes speratus*	X	X	X	Mitaka and Fujita (2021)

*Note:* Studies included here summarize caste‐associated patterns for descriptive comparison and were not necessarily part of the filtered worker dataset used for the ecological statistics.

Several studies also documented compounds or profiles associated with reproductive individuals. In *Zootermopsis nevadensis* , actively reproductive males and females contained four polyunsaturated alkenes that were absent or detected only in trace amounts in workers and soldiers (Liebig et al. 2009). In *Reticulitermes flavipes* , queens and kings contained heneicosane together with other strongly royal‐enriched hydrocarbons reported in association with royal recognition (Funaro et al. 2018). In *Reticulitermes speratus*, queens showed a markedly higher proportion of n‐pentacosane than other castes within an otherwise shared CHC background (Mitaka and Fujita 2021). In *Cryptotermes secundus*, queens differed from workers by a profile enriched in longer‐chain and more branched hydrocarbons (Weil et al. 2009). In *Nasutitermes acajutlae*, workers, soldiers, and alates shared the same broad hydrocarbon background, but soldiers were relatively enriched in early‐eluting saturated compounds, whereas workers and alates showed higher representation of unsaturated fractions.

### 3.6 Exploratory Ecological Patterns in Worker CHC Profiles

At the species level, the strongest ecological association involved life type, not feeding group or broad climate category. In the filtered species‐collapsed worker matrix, dimethylalkanes were present in only 2 of 8 drywood/enclosed wood nesters, but in 24 of 29 species representing the other life types combined. This contrast was significant in Fisher's exact test (*p* = 0.0041). Alkatrienes were present in 5 of 8 drywood species but only 6 of 29 non‐drywood species, also yielding a significant association (*p* = 0.035). When all life‐type categories were compared simultaneously, the presence of dimethylalkanes differed significantly among them (χ^2^ = 10.29, df = 3, *p* = 0.016), whereas alkatrienes, alkadienes, alkenes, and pooled unsaturated compounds did not. Class richness also did not differ among life types (Kruskal–Wallis *p* = 0.795), indicating that the ecological pattern was driven by the distribution of particular hydrocarbon classes rather than by overall chemical richness.

The family‐level sensitivity check showed that all drywood/enclosed wood nesters belonged to Kalotermitidae (Table S2); therefore, when Kalotermitidae was excluded, the drywood/non‐drywood comparison was no longer estimable. In leave‐one‐family‐out Fisher's exact tests, the dimethylalkane association remained significant after excluding Archotermopsidae (*p* = 0.0069), Mastotermitidae (*p* = 0.0049), Rhinotermitidae (*p* = 0.0062), or Termitidae (*p* = 0.0061). By contrast, the alkatriene association was less stable: it remained significant after excluding Mastotermitidae (*p* = 0.0400) or Rhinotermitidae (*p* = 0.0257), but not after excluding Archotermopsidae (*p* = 0.0789) or Termitidae (*p* = 0.0721). The dimethylalkane test remained significant in all leave‐one‐family‐out comparisons in which the contrast was estimable, whereas the alkatriene test did not. The drywood/non‐drywood contrast was not estimable after excluding Kalotermitidae because all drywood/enclosed wood nesters in the filtered dataset belonged to that family.

Broad climate category produced weaker and less stable associations. After climates were collapsed into a tropical‐lowland versus non‐tropical contrast, none of the major class presence tests remained significant at *p* < 0.05. Feeding group was not informative in the present dataset because most included taxa were wood feeders, resulting in limited ecological contrast and low statistical power for detecting feeding‐related patterns.

## 4 Discussion

The present synthesis indicates that termite CHC diversity is extensive and unevenly distributed across taxa, castes, and ecological categories. However, the available dataset remains modest, heterogeneous, and uneven in taxonomic coverage. The quantitative worker dataset included only a small fraction of known termite diversity, and the underlying studies differed in extraction procedures, chromatographic resolution, compound identification criteria, and reporting formats. In addition, ecological variables such as life type, feeding group, and climate were necessarily simplified into broad categories that may not fully capture colony‐level microclimatic conditions. Because phylogenetic relationships were not incorporated into the statistical analyses, the ecological and taxonomic patterns discussed below should be interpreted as descriptive and hypothesis‐generating rather than as formal tests of adaptive or phylogenetic mechanisms.

The patterns reported here can also be placed within a broader insect chemical ecology framework. Across insects, CHC diversity often reflects quantitative modification of shared compound classes rather than the appearance of entirely novel chemical repertoires (Johansson and Jones 2007; Steiger and Stökl 2014). In non‐eusocial insects, CHCs have been linked to mate choice, sexual selection, sex‐specific variation, mating status, and species recognition. For example, studies in the Australian field cricket *Teleogryllus oceanicus* showed sexual selection on male CHC profiles and demonstrated that CHCs can influence female attractiveness to males (Thomas and Simmons 2009, 2010). In sagebrush crickets, CHCs have also been examined as sexually selected traits under field conditions (Steiger et al. 2013), while recent work on *Isophya* bush crickets showed that CHC profiles vary according to species identity, sex, and mating status (Sevgili et al. 2025). In this context, termite CHC diversity appears to represent a social‐insect example of a wider pattern in which ecological, reproductive, and taxonomic differences are often expressed through changes in the relative abundance of recurring CHC classes. Because *Mastotermes* is the basalmost living termite, a chemically narrow or obviously primitive profile might have been expected. Instead, *Mastotermes darwiniensis* shows one of the broadest known worker blends, with substantial fractions of n‐alkanes, n‐alkenes, alkadienes, and monomethylalkanes. This result does not fit a model in which early termite chemistry was chemically reduced, and later lineages became progressively more elaborate. Rather, it is compatible with the hypothesis that early termite CHC repertoires may have been chemically versatile. This interpretation is consistent with the broader Blattodea‐scale results of Golian et al. (2024), who found that chemical divergence does not mirror phylogeny or social complexity in a straightforward way.

The more polarized profiles observed in several extant termites may represent taxon‐specific chemical specializations. *Coptotermes formosanus* appears as a lineage strongly shifted toward monomethylalkane dominance with little or no detectable unsaturated contribution, whereas *Parvitermes wolcotti* represents a highly unsaturated worker condition dominated by alkatrienes. Between these poles lie taxa such as *Reticulitermes flavipes* , *Neotermes connexus* , and *Mastotermes darwiniensis*, in which substantial contributions from multiple classes are retained. This range of profiles is more consistent with repeated taxon‐specific reweighting of a shared chemical toolkit than with repeated replacement of one chemical regime by another.

The genus‐level patterns reinforce the same point. Kalotermitidae do not conform to a single chemical syndrome. Some genera, such as *Cryptotermes* and *Incisitermes*, are frequently rich in unsaturated compounds, whereas others, such as *Pterotermes*, are dominated by saturated and monomethyl‐branched fractions. Termitidae are similarly heterogeneous, including dimethyl‐rich *Nasutitermes* species and highly unsaturated *Parvitermes*. Even within Rhinotermitidae, the contrast between strongly methyl‐branched *Coptotermes* and the more compositionally mixed *Reticulitermes* shows that substantial divergence may arise within the same higher lineage. These patterns show that dominant worker chemistry varies among sampled genera and families, without requiring interpretation as a single directional trend.

The exploratory ecological analyses identified an association between nesting life type and worker CHC composition in the literature‐derived dataset. Drywood/enclosed wood nesters showed a lower frequency of dimethylalkanes and a higher frequency of alkatrienes than the other life types, while overall class richness did not differ among life types. However, the sensitivity check showed that all drywood/enclosed wood nesters in the filtered dataset belonged to Kalotermitidae; therefore, this association may reflect nesting life type, broad family‐level structure, or both. A possible ecological explanation is that nesting life type may influence the balance between waterproofing and communication functions of CHCs. Drywood termites live within wooden substrates, whereas subterranean, dampwood, and mound‐associated termites may differ in soil contact, humidity exposure, nest buffering, and exposure to external environmental variation. These ecological differences could influence CHC composition because straight‐chain n‐alkanes generally pack more tightly and have higher melting temperatures than unsaturated or methyl‐branched hydrocarbons, properties expected to increase cuticular viscosity and reduce permeability (Gibbs and Pomonis 1995; Menzel et al. 2019). By contrast, methyl branching and unsaturation disrupt molecular packing, lower melting temperature, and modify the physical properties of the cuticular layer, while also expanding the potential signal space of CHC blends (Gibbs and Pomonis 1995; Blomquist and Bagnères 2010; Menzel et al. 2019). Unsaturated hydrocarbons may also be more chemically labile under environmental exposure (Sprenger et al. 2020).

Experimental and comparative studies provide a rationale for considering climate and microclimate in future work, although the present dataset did not recover a strong climate‐only signal. In *Cryptotermes brevis* , Woodrow et al. (2000) showed experimentally that both temperature and relative humidity can modify cuticular hydrocarbon composition, with temperature exerting particularly strong effects on the abundance of individual compounds. Similar environmentally induced shifts are known from other insects, where temperature, humidity, and desiccation regime influence the balance of saturated, unsaturated, and branched hydrocarbons (Menzel et al. 2017, 2019). The weak climate signal in the current analysis should therefore not be interpreted as evidence that climate is irrelevant. More likely, broad regional climate categories are simply too coarse to capture the conditions actually experienced by the termite cuticle.

The caste‐level literature adds a second important layer to this picture. Across the reviewed studies, workers, soldiers, nymphs, and reproductives usually share the same major hydrocarbon classes, and caste differences most often arise through shifts in relative abundance rather than through complete chemical turnover. This is particularly clear in *Reticulitermes virginicus* and *Zootermopsis nevadensis* , and it is broadly consistent with the patterns reported for *Nasutitermes acajutlae*, *Coptotermes formosanus* , and *C. gestroi* (Howard et al. 1982; Haverty et al. 1996; Sevala et al. 2000; Gordon et al. 2020). These results suggest that termite caste chemistry is generally built on a shared colony‐level background rather than on wholly caste‐exclusive chemical repertoires.

At the same time, reproductives in several taxa also show additional caste‐associated compounds or strongly biased reproductive profiles. This is evident in *Zootermopsis nevadensis* , where active reproductives contain polyunsaturated compounds absent or nearly absent from workers and soldiers (Liebig et al. 2009), in *Reticulitermes flavipes* , where royal recognition is associated with heneicosane and other royal‐enriched hydrocarbons (Funaro et al. 2018), and in *Cryptotermes secundus*, where queens differ from workers by enrichment in longer‐chain and more strongly branched compounds (Weil et al. 2009). These cases indicate that baseline worker chemistry and caste‐specific signaling do not necessarily evolve along the same axis. A lineage may remain methyl‐branched dominated at the worker level while still deploying unsaturated or caste‐biased compounds in a reproductive context.

Taken together, this synthesis shows that termite CHC profiles are chemically diverse, that caste differentiation is often expressed through quantitative shifts within broadly shared hydrocarbon repertoires, and that worker CHC composition shows exploratory associations with nesting life type in the available literature‐derived dataset. These findings support termite CHCs as a useful system for examining how cuticular chemistry varies across social, ecological, and taxonomic contexts. Future progress will depend on denser sampling of basal and understudied termite taxa, more consistent caste‐resolved quantification, compound‐level data on chain length and branching position, and more explicit integration of ecological exposure, nest microclimate, and comparative evolutionary methods.

## Author Contributions

**V. Palma‐Onetto:** conceptualization (lead), data curation (lead), formal analysis (lead), investigation (lead), supervision (lead), visualization (equal), writing – original draft (lead), writing – review and editing (lead). **J. Cabrera‐Riquelme:** conceptualization (supporting), data curation (supporting), methodology (supporting), resources (supporting), validation (supporting), visualization (equal), writing – review and editing (supporting). **S. Ahmad:** conceptualization (supporting), visualization (supporting), writing – review and editing (equal).

## Funding

This work was supported by Coordenação de Aperfeiçoamento de Pessoal de Nível Superior. Gender Observatory of Universidad Técnica Federico Santa María.

## Conflicts of Interest

The authors declare no conflicts of interest.

## Supporting information


**Figure S1:** PRISMA flow diagram summarizing study identification, screening, eligibility assessment, and final inclusion.
**Table S1:** Species‐by‐study dataset of termite cuticular hydrocarbon profiles used in the comparative synthesis. Includes taxonomic information, caste or worker‐equivalent stage, locality, ecological variables, major cuticular hydrocarbon classes, and available relative abundance data extracted from the reviewed literature.
**Table S2:** Data for family‐level sensitivity analysis of the association between nesting life type and worker CHC classes. Summarizes leave‐one‐family‐out Fisher's exact tests for the drywood/enclosed wood versus non‐drywood contrast in dimethylalkanes and alkatrienes.

## Data Availability

The data supporting the findings of this study are available within the article and its Supporting Information.
