# Supplementary material for: Comparative Patterns of Cuticular Hydrocarbon Diversity in Termites Across Castes and Nesting Life Types
Source: Ecol Evol. 2026 Aug 9;16(8):e74070. doi: 10.1002/ece3.74070 (PMC13454219; doi:10.1002/ece3.74070)
Supplement: Supplementary file 1 [file ECE3-16-e74070-s001.pdf]

Supplementary material

Figure S1. PRISMA flow diagram summarizing study identification, screening, eligibility assessment, and final inclusion.

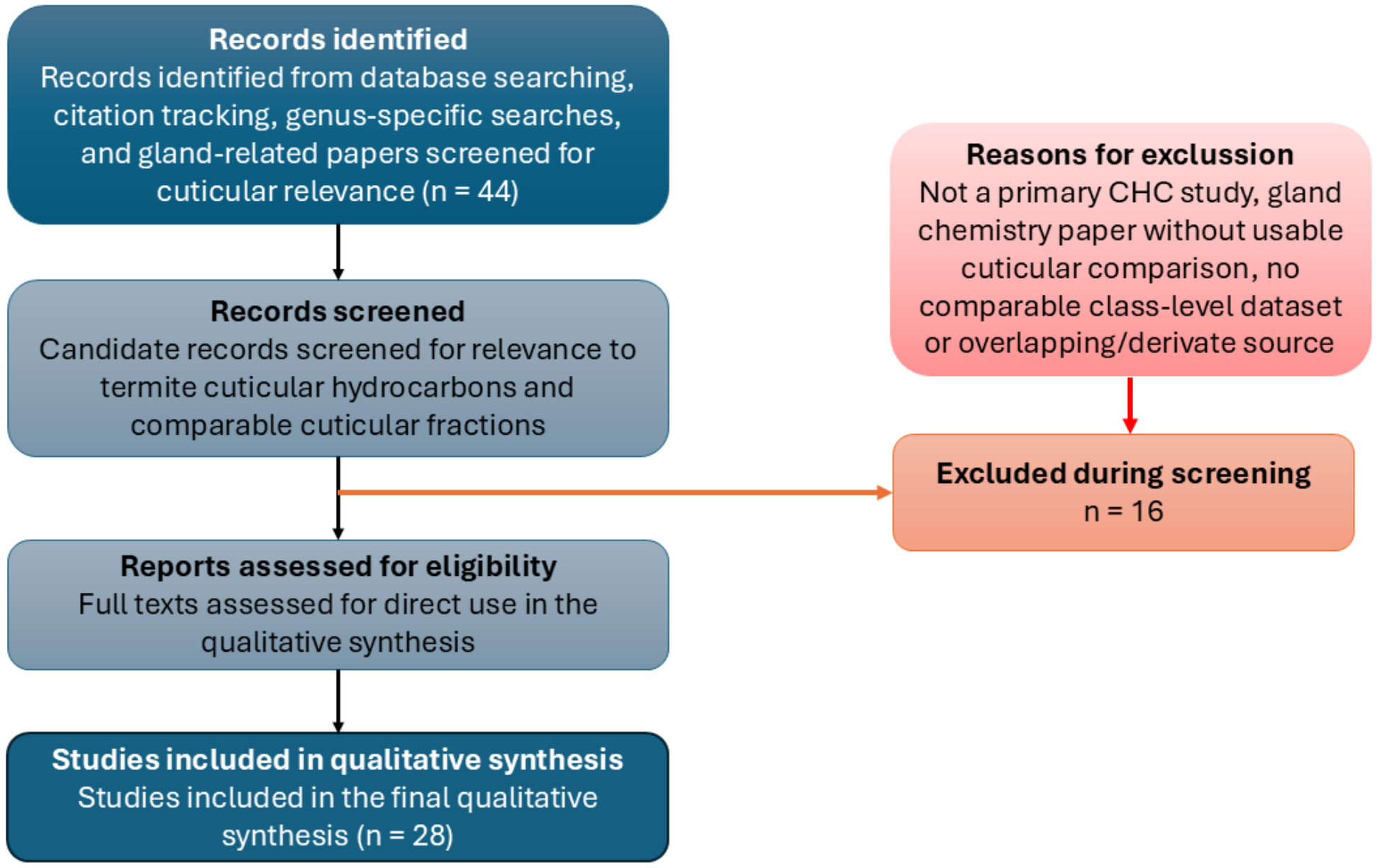

Table S1. Species-by-study dataset of termite cuticular hydrocarbon profiles used in the comparative synthesis. Compounds were standardized into broad CHC classes to allow comparison across studies with different analytical resolution and reporting formats.

Family	Genus	Species	Feeding group	Life type	Climate	City	Study	n-Alkanes	Alkenes	Alkadienes	Alkatrienes	Monomethylalkanes	Dimethylalkanes	Trimethylalkanes	Unresolved branched
Kalotermitidae	Neotermes	chilensis	Wood feeder	Drywood	Mediterranean	Santiago (Chile)	Aguilera-Olivares et al. 2016	Yes	Yes			Yes			
Rhinotermitidae	Reticulitermes	flavipes	Wood feeder	Subterranean	Subtropical	Georgia (USA)	Bagnères et al. 1990	Yes	Yes	Yes		Yes	Yes		Yes
Rhinotermitidae	Reticulitermes	santonensis	Wood feeder	Subterranean	Temperate	Charente (France)	Bagnères et al. 1990	Yes	Yes	Yes		Yes	Yes		Yes
Rhinotermitidae	Reticulitermes	banyulensis	Wood feeder	Subterranean	Mediterranean	Southwestern France (not specified)	Bagnères et al. 1991	Yes	Yes			Yes	Yes		Yes
Rhinotermitidae	Reticulitermes	grassei	Wood feeder	Subterranean	Mediterranean	Italy (city not specified)	Bagnères et al. 1991	Yes	Yes			Yes	Yes		Yes
Rhinotermitidae	Heterotermes	tenuis	Wood feeder	Subterranean	Subtropical	Sao Paulo (Brazil)	Batista-Pereira et al. 2004	Yes							
Termitidae	Nasutitermes	coxipoensis	Wood feeder	Mound	Mesothermal highland	Serrá fo Cipó (Brazil)	Camarota et al. 2025	Yes				Yes	Yes	Yes	
Rhinotermitidae	Reticulitermes	flavipes	Wood feeder	Subterranean	Subtropical	Raleigh (USA)	Funaro et al. 2018 PNAS	Yes		Yes	Yes	Yes	Yes		Yes
Mastotermitidae	Mastotermes	darwiniensis	Wood feeder	Dampwood	Tropical monsoon	Australia	Golian et al. 2024	Yes	Yes	Yes		Yes	Yes	Yes	
Rhinotermitidae	Coptotermes	gestroi	Wood feeder	Subterranean	Tropical monsoon	Florida	Gordon et al. 2020	Yes				Yes			Yes

Rhinotermitidae	Reticulitermes	sp.	Wood feeder	Subterranean	Temperate to semi-arid	Arizona and Utah (USA)	Haverty & Nelson 2007		Yes	Yes	Yes		Yes		Yes
Archotermopsidae	Zootermopsis	angusticollis	Wood feeder	Dampwood	Mediterranean	California (USA)	Haverty et al. 1988	Yes	Yes			Yes	Yes		Yes
Archotermopsidae	Zootermopsis	laticeps	Wood feeder	Dampwood	Semi-arid steppe	Arizona (USA)	Haverty et al. 1988	Yes	Yes			Yes	Yes		Yes
Archotermopsidae	Zootermopsis	nevadensis	Wood feeder	Dampwood	Mediterranean	California (USA)	Haverty et al. 1988	Yes	Yes				Yes	Yes	Yes
Termitidae	Nasutitermes	costalis	Wood feeder	Mound	Tropical rainforest	Caribe (not specified)	Haverty et al. 1990a	Yes	Yes			Yes	Yes	Yes	
Termitidae	Nasutitermes	ephratae	Wood feeder	Mound	Tropical rainforest	Caribe (not specified)	Haverty et al. 1990a	Yes	Yes			Yes	Yes	Yes	
Rhinotermitidae	Coptotermes	formosanus	Wood feeder	Subterranean	Tropical island	Hawai	Haverty et al. 1990b	Yes				Yes	Yes	Yes	Yes
Termitidae	Nasutitermes	acajutlae	Wood feeder	Mound	Tropical rainforest	Caribe (not specified)	Haverty et al. 1996	Yes	Yes	Yes	Yes	Yes			
Termitidae	Nasutitermes	acajutlae	Wood feeder	Mound	Tropical island	BVI Islands	Haverty et al. 1997	Yes	Yes	Yes	Yes	Yes			
Kalotermitidae	Procryptotermes	corniceps	Wood feeder	Drywood	Tropical island	BVI Islands	Haverty et al. 1997	Yes	Yes	Yes	Yes	Yes			
Termitidae	Nasutitermes	costalis	Wood feeder	Mound	Tropical island	BVI Islands	Haverty et al. 1997	Yes	Yes			Yes	Yes	Yes	
Kalotermitidae	Neotermes	mona	Wood feeder	Dampwood	Tropical island	BVI Islands	Haverty et al. 1997	Yes	Yes			Yes	Yes		
Termitidae	Parvitermes	wolcotti	Wood feeder	Dampwood	Tropical island	BVI Islands	Haverty et al. 1997	Yes	Yes	Yes	Yes	Yes			
Kalotermitidae	Cryptotermes	brevis	Wood feeder	Drywood	Tropical island	Hawai	Haverty et al. 1997; Haverty et al. 2000	Yes	Yes	Yes	Yes	Yes	Yes		Yes
Kalotermitidae	Cryptotermes	cynocephalus	Wood feeder	Drywood	Tropical island	Hawai	Haverty et al. 2000	Yes	Yes	Yes	Yes	Yes			Yes
Rhinotermitidae	Coptotermes	formosanus	Wood feeder	Subterranean	Subtropical	Hawai	Haverty et al. 2000	Yes				Yes	Yes	Yes	Yes
Rhinotermitidae	Coptotermes	vastator	Wood feeder	Subterranean	Tropical island	Hawai	Haverty et al. 2000	Yes				Yes	Yes		Yes
Kalotermitidae	Neotermes	connexus	Wood feeder	Drywood	Tropical island	Hawai	Haverty et al. 2000; Haverty & Nelson 2005	Yes	Yes	Yes	Yes	Yes			
Kalotermitidae	Incisitermes	immigrans	Wood feeder	Drywood	Tropical island	Hawai	Haverty et al. 2000	Yes	Yes	Yes	Yes	Yes			
Kalotermitidae	Incisitermes	minor	Wood feeder	Drywood	Tropical island	Hawai	Haverty et al. 2000	Yes	Yes	Yes		Yes	Yes		
Rhinotermitidae	Reticulitermes	virginicus	Wood feeder	Subterranean	Subtropical	Mississippi (USA)	Howard et al. 1982	Yes	Yes	Yes			Yes		Yes
Termitidae	Nasutitermes	corniger	Wood feeder	Mound	Tropical rainforest	Several cities (Panamá)	Howard et al. 1988	Yes	Yes			Yes	Yes	Yes	
Termitidae	Nasutitermes	ephratae	Wood feeder	Mound	Tropical rainforest	Several cities (Panamá)	Howard et al. 1988	Yes	Yes			Yes	Yes	Yes	
Rhinotermitidae	Reticulitermes	santonensis	Wood feeder	Subterranean	Mediterranean	Paris (France)	Jenkins et al. 2000 / Dronnet et al. 2006	Yes	Yes						Yes
Termitidae	Macrotermes	falciger	Fungus grower	Mound	Savanna	Africa (city not specified)	Kaib et al. 2002	Yes	Yes	Yes			Yes		Yes
Termitidae	Macrotermes	subhyalinus	Fungus grower	Mound	Wet savanna	Comoé (Ivory Coast)	Kaib et al. 2004	Yes	Yes	Yes		Yes	Yes		Yes
Termitidae	Macrotermes	falciger	Fungus grower	Mound	Savanna	Africa (city not specified)	Marten et al. 2009	Yes	Yes	Yes			Yes		Yes
Termitidae	Macrotermes	herus	Fungus grower	Mound	Savanna	Africa (city not specified)	Marten et al. 2009	Yes	Yes	Yes			Yes		Yes
Termitidae	Macrotermes	jeanneli	Fungus grower	Mound	Savanna	Africa (city not specified)	Marten et al. 2009	Yes	Yes	Yes			Yes		Yes
Termitidae	Macrotermes	subhyalinus	Fungus grower	Mound	Savanna	Comoé (Ivory Coast)	Marten et al. 2009	Yes	Yes	Yes			Yes		Yes
Rhinotermitidae	Reticulitermes	speratus	Wood feeder	Subterranean	Subtropical	Several cities (Japan)	Mitaka & Fujita 2021	Yes				Yes			Yes
Rhinotermitidae	Reticulitermes	speratus	Wood feeder	Subterranean	Subtropical	Kyoto (Japan)	Mitaka & Matsuura 2020	Yes							Yes
Rhinotermitidae	Reticulitermes	hesperus	Wood feeder	Subterranean	Mediterranean	California (USA)	Nelson et al. 2008	Yes	Yes	Yes	Yes	Yes	Yes	Yes	
Rhinotermitidae	Reticulitermes	flavipes	Wood feeder	Subterranean	Subtropical	Florida and Louisiana (USA)	Perdereau et al. 2010		Yes						Yes
Archotermopsidae	Zootermopsis	nevadensis	Wood feeder	Dampwood	Mediterranean	California (USA)	Sevala et al. 2000	Yes	Yes			Yes	Yes	Yes	
Rhinotermitidae	Reticulitermes	speratus	Wood feeder	Subterranean	Subtropical	Kyoto (Japan)	Takahashi & Grassa 1995	Yes	Yes						Yes
Rhinotermitidae	Reticulitermes	lucifugus	Wood feeder	Subterranean	Mediterranean	Italy (multiple cities)	Uva et al. 2004	Yes	Yes	Yes	Yes	Yes	Yes		Yes
Rhinotermitidae	Heterotermes	brevicatena	Wood feeder	Subterranean	Temperate to semi-arid	Australia	Watson et al. 1989	Yes	Yes	Yes		Yes	Yes		

Rhinotermitidae	Heterotermes	ferox	Wood feeder	Subterranean	Subtropical	Australia	Watson et al. 1989	Yes			Yes	Yes	Yes		
Rhinotermitidae	Heterotermes	longicatena	Wood feeder	Subterranean	Temperate to semi-arid	Australia	Watson et al. 1989	Yes	Yes	Yes		Yes			
Kalotermitidae	Cryptotermes	secundus	Wood feeder	Drywood	Tropical savanna	Australia	Weil et al. 2009	Yes	Yes	Yes		Yes			Yes

Table S2. Family-level sensitivity analysis of the association between nesting life type and worker CHC classes. The table summarizes leave-one-family-out Fisher's exact tests for the drywood/enclosed wood versus non-drywood contrast in dimethylalkanes and alkatrienes. For each analysis, one termite family was excluded and the drywood/non-drywood association was recalculated. NA indicates that the test was not estimable because all drywood/enclosed wood nesters in the filtered dataset belonged to Kalotermitidae.

Excluded family	Trait	N after exclusion	Drywood N	Non-drywood N	Drywood present	Non-drywood present	Odds ratio	Fisher p
Archotermopsidae	Dimethylalkanes	34	8	26	2	21	0,0794	0,0069
Archotermopsidae	Alkatrienes	34	8	26	5	6	5,5556	0,0789
Kalotermitidae	Dimethylalkanes	28	0	28				
Kalotermitidae	Alkatrienes	28	0	28				
Mastotermitidae	Dimethylalkanes	36	8	28	2	23	0,0725	0,0049
Mastotermitidae	Alkatrienes	36	8	28	5	6	6,1111	0,0400
Rhinotermitidae	Dimethylalkanes	23	8	15	2	13	0,0513	0,0062
Rhinotermitidae	Alkatrienes	23	8	15	5	2	10,8333	0,0257
Termitidae	Dimethylalkanes	27	8	19	2	16	0,0625	0,0061
Termitidae	Alkatrienes	27	8	19	5	4	6,2500	0,0721

References

Aguilera-Olivares, D., Flores-Prado, L., Véliz, D., & Niemeyer, H. M. (2015). Mechanisms of inbreeding avoidance in the one-piece drywood termite *Neotermes chilensis.* Insectes Sociaux, 62(2), 237-245.

Bagnères, A. G., Clément, J. L., Blum, M. S., Severson, R. F., Joulie, C., & Lange, C. (1990). Cuticular hydrocarbons and defensive compounds of *Reticulitermes flavipes* (Kollar) and *R. santonensis* (Feytaud): polymorphism and chemotaxonomy. Journal of Chemical Ecology, 16(12), 3213-3244. https://doi.org/10.1007/BF00982094

Bagnères, A. G., Killian, A., Clement, J. L., & Lange, C. (1991). Interspecific recognition among termites of the genus *Reticulitermes*: evidence for a role for the cuticular hydrocarbons. Journal of Chemical Ecology, 17(12), 2397-2420. https://doi.org/10.1007/BF00994590

Batista-Pereira, L. G., dos Santos, M. G., Corrêa, A. G., Fernandes, J. B., Arab, A., Costa-Leonardo, A. M., Dietrich, C. R. R. C., Pereira, D. A., & Bueno, O. C. (2004). Cuticular hydrocarbons of *Heterotermes tenuis* (Isoptera: Rhinotermitidae): Analyses and electrophysiological studies. Zeitschrift für Naturforschung C, 59(1-2), 135-139. https://doi.org/10.1515/znc-2004-1-226

Camarota, F., Viana-Junior, A. B., Vidal, D. M., Zarbin, P. H., & Neves, F. S. (2025). Changes in the cuticular profile but not aggression of termites along a tropical elevation gradient. Ecological Entomology, 50(1), 74-83. https://doi.org/10.1111/een.13379

Dronnet, S., Lohou, C., Christides, J. P., & Bagnères, A. G. (2006). Cuticular hydrocarbon composition reflects genetic relationship among colonies of the introduced termite *Reticulitermes santonensis* Feytaud. Journal of Chemical Ecology, 32(5), 1027-1042. https://doi.org/10.1007/s10886-006-9043-x

Haverty, M. I., Thorne, B. L., & Page, M. (1990a). Surface hydrocarbon components of two species of *Nasutitermes* from Trinidad. Journal of chemical ecology, 16(8), 2441-2450.

Haverty, M. I., Nelson, L. J., & Page, M. (1990b). Cuticular hydrocarbons of four populations of *Coptotermes formosanus* Shiraki in the United States: Similarities and origins of introductions. Journal of chemical ecology, 16(5), 1635-1647.

Haverty, M. I., Page, M., Nelson, L. J., & Blomquist, G. J. (1988). Cuticular hydrocarbons of dampwood termites, *Zootermopsis*: Intra- and intercolony variation and potential as taxonomic characters. Journal of Chemical Ecology, 14, 1035-1058. https://doi.org/10.1007/BF01018791

Haverty, M. I., Thorne, B. L., & Nelson, L. J. (1996). Hydrocarbons of *Nasutitermes acajutlae* and comparison of methodologies for sampling cuticular hydrocarbons of Caribbean termites for taxonomic and ecological studies. Journal of Chemical Ecology, 22, 2081-2109. https://doi.org/10.1007/BF02040096

Haverty, M. I., Collins, M. S., Nelson, L. J., & Thorne, B. L. (1997). Cuticular hydrocarbons of termites of the British Virgin Islands. Journal of Chemical Ecology, 23, 927-964. https://doi.org/10.1023/B:JOEC.0000006381.75185.86

Haverty, M. I., Woodrow, R. J., Nelson, L. J., & Grace, J. K. (2000). Cuticular hydrocarbons of termites of the Hawaiian Islands. Journal of Chemical Ecology, 26, 1167-1191. https://doi.org/10.1023/A:1005479826651

Haverty, M. I., & Nelson, L. J. (2005). Identification of termite species by the hydrocarbons in their feces. Journal of Chemical Ecology, 31, 2119-2151. https://doi.org/10.1007/s10886-005-6081-8

Haverty, M. I., & Nelson, L. J. (2007). *Reticulitermes* (Isoptera: Rhinotermitidae) in Arizona: Multiple cuticular hydrocarbon phenotypes indicate additional taxa. Annals of the Entomological Society of America, 100(2), 206–221. https://doi.org/10.1603/0013-8746(2007)100[206:RIRIAM]2.0.CO;2

Howard, R. W., McDaniel, C. A., Nelson, D. R., Blomquist, G. J., Gelbaum, L. T., & Zalkow, L. H. (1982). Cuticular hydrocarbons of *Reticulitermes virginicus* (Banks) and their role as potential species- and caste-recognition cues. Journal of Chemical Ecology, 8, 1227-1239. https://doi.org/10.1007/BF00990755

Howard, R. W., Thorne, B. L., Levings, S. C., & McDaniel, C. A. (1988). Cuticular hydrocarbons as chemotaxonomic characters for *Nasutitermes corniger* (Motschulsky) and *N. ephratae* (Holmgren) (Isoptera: Termitidae). Annals of the Entomological Society of America, 81(3), 395–399. https://doi.org/10.1093/aesa/81.3.395

Jenkins, T. M., Haverty, M. I., Basten, C. J., Nelson, L. J., Page, M., & Forschler, B. T. (2000). Correlation of mitochondrial haplotypes with cuticular hydrocarbon phenotypes of sympatric *Reticulitermes* species from the southeastern United States. Journal of Chemical Ecology, 26, 1525–1542. https://doi.org/10.1023/A:1005548111591

Kaib, M., Franke, S., Francke, W., & Brandl, R. (2002). Cuticular hydrocarbons in a termite: Phenotypes and a neighbour-stranger effect. Physiological Entomology, 27(3), 189-198. https://doi.org/10.1046/j.1365-3032.2002.00292.x

Kaib, M., Jmhasly, P., Wilfert, L., Durka, W., Franke, S., Francke, W., Leuthold, R. H., & Brandl, R. (2004). Cuticular hydrocarbons and aggression in the termite *Macrotermes subhyalinus*. Journal of Chemical Ecology, 30(2), 365–385. https://doi.org/10.1023/B:JOEC.0000017983.89279.c5

Marten, A., Kaib, M., & Brandl, R. (2009). Cuticular hydrocarbon phenotypes do not indicate cryptic species in fungus-growing termites (Isoptera: Macrotermitinae). Journal of Chemical Ecology, 35(5), 572–579. https://doi.org/10.1007/s10886-009-9626-4

Mitaka, Y., & Matsuura, K. (2020). Age-dependent increase in soldier pheromone of the termite *Reticulitermes speratus*. Journal of Chemical Ecology, 46(5–6), 483–489. https://doi.org/10.1007/s10886-020-01182-6

Mitaka, Y., & Fujita, T. (2021). Cuticular hydrocarbon profile for queen recognition in the termite *Reticulitermes speratus*. Research Square. https://doi.org/10.21203/rs.3.rs-490573/v1

Nelson, L. J., Haverty, M. I., & Page, M. (2008). Cuticular hydrocarbons of *Reticulitermes* (Isoptera: Rhinotermitidae) from northern California indicate species and population differences. Journal of Chemical Ecology, 34, 1351–1364. https://doi.org/10.1007/s10886-008-9543-0

Perdereau, E., Bagnères, A.-G., Bankhead-Dronnet, S., Dupont, S., Zimmermann, M., & Vargo, E. L. (2010). Global genetic analysis reveals the invasive history of the termite *Reticulitermes flavipes*. Biological Invasions, 12, 1477–1489. https://doi.org/10.1007/s10530-009-9553-0

Sevala, V. L., Bagnères, A.-G., & Schal, C. (2000). Cuticular hydrocarbons of the dampwood termite *Zootermopsis nevadensis*: Caste differences and role of lipophorin in transport of hydrocarbons and hydrocarbon metabolites. Journal of Chemical Ecology, 26, 765-789. https://doi.org/10.1023/A:1005440624678

Takahashi, S., & Gassa, A. (1995). Roles of cuticular hydrocarbons in intra- and interspecific recognition behavior of two Rhinotermitidae species. Journal of Chemical Ecology, 21(11), 1837–1845. https://doi.org/10.1007/BF02033680

Uva, P., Clément, J.-L., & Bagnères, A.-G. (2004). Colonial and geographic variations in agonistic behaviour, cuticular hydrocarbons and mtDNA of Italian populations of *Reticulitermes lucifugus* (Isoptera, Rhinotermitidae). Insectes Sociaux, 51, 163–170. https://doi.org/10.1007/s00040-003-0728-7

Watson, J. A. L., Brown, W. V., Miller, L. R., Carter, F. L., & Lacey, M. J. (1989). Taxonomy of *Heterotermes* (Isoptera: Rhinotermitidae) in south-eastern Australia: cuticular hydrocarbons of workers, and soldier and alate morphology. Systematic Entomology, 14(3), 299-325. https://doi.org/10.1111/j.1365-3113.1989.tb00287.x

Weil, T., Hoffmann, K., Kroiss, J., Strohm, E., & Korb, J. (2009). Scent of a queen-cuticular hydrocarbons specific for female reproductives in lower termites. Naturwissenschaften, 96, 315-319. https://doi.org/10.1007/s00114-008-0475-8
